# lncRNAs Functioned as ceRNA to Sponge miR-15a-5p Affects the Prognosis of Pancreatic Adenocarcinoma and Correlates With Tumor Immune Infiltration

**DOI:** 10.3389/fgene.2022.874667

**Published:** 2022-07-11

**Authors:** Yu Wang, Zhen Wang, KaiQiang Li, WeiLing Xiang, BinYu Chen, LiQin Jin, Ke Hao

**Affiliations:** ^1^ Laboratory Medicine Center, Allergy Center, Department of Transfusion Medicine, Zhejiang Provincial People’s Hospital (Affiliated People’s Hospital, Hangzhou Medical College), Hangzhou, Zhejiang, China; ^2^ School of Laboratory Medicine and Life Science, Wenzhou Medical University, Wenzhou, Zhejiang, China; ^3^ Department of Scientific Research, Zhejiang Provincial People’s Hospital, People’s Hospital of Hangzhou Medical College, Hangzhou, Zhejiang, China

**Keywords:** pancreatic adenocarcinoma, ceRNA network, immune infiltration, therapeutic targets, prognosis

## Abstract

Pancreatic adenocarcinoma (PAAD) is one of the most common malignant tumors with poor prognosis worldwide. Mounting evidence suggests that the expression of lncRNAs and the infiltration of immune cells have prognostic value for patients with PAAD. We used Gene Expression Omnibus (GEO) database and identified six genes (COL1A2, ITGA2, ITGB6, LAMA3, LAMB3, and LAMC2) that could affect the survival rate of pancreatic adenocarcinoma patients. Based on a series of *in silico* analyses for reverse prediction of target genes associated with the prognosis of PAAD, a ceRNA network of mRNA (COL1A2, ITGA2, LAMA3, LAMB3, and LAMC2)–microRNA (miR-15a-5p)–long non-coding RNA (LINC00511, LINC01578, PVT1, and TNFRSF14-AS1) was constructed. We used the algorithm “CIBERSORT” to assess the proportion of immune cells and found three overall survival (OS)–associated immune cells (monocytes, M1 macrophages, and resting mast cell). Moreover, the OS-associated gene level was significantly positively associated with immune checkpoint expression and biomarkers of immune cells. In summary, our results clarified that ncRNA-mediated upregulation of OS-associated genes and tumor-infiltration immune cells (monocytes, M1 macrophages M1, and resting mast cell resting) correlated with poor prognosis in PAAD.

## Introduction

Pancreatic adenocarcinoma (PAAD) is one of the malignant tumors of the digestive system, and it is difficult to diagnose and treat (Siegel et al., 2018). Due to insufficient early symptoms, more than 50% of PAAD patients have metastatic disease at diagnosis (Balsano et al., 2019). Despite the significant improvement in diagnostic imaging and surgical mortality in the past 20 years, the 5-year survival rate is still less than 5% (Philip et al., 2009). The treatment options for pancreatic cancer are limited. About 80% of patients with PAAD lose the chance of surgical resection (Wang et al., 2020). In addition, patients undergoing complete tumor resection usually have a local or distant recurrence within 2 years after the surgery (Hessmann et al., 2020). Other treatment strategies such as radiation, combination chemotherapy, and biological therapy also have limited efficacy (Iacobuzio-Donahue et al., 2009; Pipas et al., 2012). The most fundamental reason is that the pathogenesis and prognostic markers of PAAD are not clear. For better performing early diagnosis, treatment, and prognostic evaluation of PAAD, it is particularly important to find effective biomarkers.

With advances in high-throughput sequencing technology, more biomarkers can be discovered to determine prognosis and improve treatment strategies (Zhang et al., 2018; Ping et al., 2020; Qiu et al., 2020). Non-coding RNAs (ncRNAs) have an important role in the occurrence and development of cancer and are promising biomarkers and therapeutic targets (Cai et al., 2020; Mu et al., 2020; Tseng et al., 2020). lncRNAs are broadly defined as non-coding RNA with a length greater than 200 nucleotides, which previously were thought to have limited protein-coding ability (Mi et al., 2020). However, accumulating evidence demonstrated that specific lncRNAs are related to cancer recurrence, progression, and metastasis (Lin et al., 2020a; Zhou et al., 2020a; Peng et al., 2020; Teng et al., 2020). Salmena et al. proposed the competitive endogenous RNA (ceRNA) hypothesis that lncRNA can affect mRNA through competitively combining different miRNAs (Salmena et al., 2011). miRNAs are a type of small single-stranded ncRNA with regulatory functions, with a length of 21–24 nucleotides (Zhang et al., 2020a; Lin et al., 2020b; Zhou et al., 2020b). One hypothesis of ceRNA considers that miRNAs can regulate multiple target genes, and the same target genes can be regulated by different miRNAs. Also, the ceRNA hypothesis also illustrates that the expression levels of lncRNA and miRNA are negatively correlated and positively correlated with mRNA expression (Wang and Liu, 2017; Li et al., 2020). Previous studies have focused on elucidating the involvement of the ceRNA network in pancreatic adenocarcinoma progression, metastasis, and prognosis, such as LINC00958 and TP73-AS1 (Chen et al., 2019; Miao et al., 2021). In addition, tumor cells and aggressive immune cells participate in the occurrence and development of cancer (Hanahan and Weinberg, 2011). It has become clear that evaluating the type and degree of tumor-infiltrating immune cells is very important for predicting progression and prognosis. However, only a few studies focus on the interaction mechanism between ceRNA networks and immune infiltrating cells. Therefore, a deeper understanding of ceRNA networks and pancreatic adenocarcinoma immune infiltrating cells is needed.

In this study, we established a significant ceRNA network that involved four lncRNAs (LINC00511, LINC01578, PVT1, and TNFRSF14-AS1), one miRNA (hsa-miR-15a-5p), and six mRNAs (COL1A2, ITGA2, ITGB6, LAMA3, LAMB3, and LAMC2). Moreover, we used CIBERSORT to infer the proportion of immune cells in pancreatic adenocarcinoma samples (Chen et al., 2018). In addition, we determined the relationship of OS-associated gene expression with biomarkers of immune cells and immune checkpoints in PAAD.

## Materials and Methods

### Data Collection and Differential Gene Expression Analysis

The four pancreatic cancer gene chip data used in this study (GSE63111, GSE23397, GSE62165, and GSE43795) were derived from the NCBI-GEO database (https://www.ncbi.nlm.nih.gov/geo). Among them, GSE63111 was included in 35 cases, including in seven tumor samples and 28 non-tumor samples; GSE23397 was included in 21 cases, including in 15 tumor samples and six non-tumor samples; GSE62165 was included in 131 cases, including in 118 tumor samples and 13 non-tumor samples; GSE43795 was included in 31 cases, including in 26 tumor samples and five non-tumor samples. We used the limma package in the R software to screen differentially expressed genes (DEGs) (Ritchie et al., 2015). The screening threshold was set to *p* < 0.05, and the absolute value of log-fold change | log2FC| ≥ 2. We used the online tool “Venny” to construct the Venn diagram of the DEGs and identified common DEGs (Oliveros, 2007-2015).

### Functional Enrichment Analysis, Interaction Network Analysis, and Hub Gene Identification

To further elucidate the potential functional annotation and pathway enrichment related to DEGs, we used the clusterProfiler package to perform the Kyoto Encyclopedia of Genes and Genomes (KEGG) and Gene Ontology (GO) enrichment analyses (Yu et al., 2012). Significance was defined as *p* < 0.05. We used an online database Search Tool for the Retrieval of Interacting Genes (STRING, https://string-db.org/) to construct the protein–protein interaction (PPI) network of DEGs, and the confidence score ≥0.9 was set as the threshold. We removed protein nodes that did not interact with other proteins. In addition, we used Cytoscape v.3.8.2 to analyze the PPI network for screening the hub genes. The CytoHubba (version: 0.1) plug-in was used to identify hub genes in the PAAD.

### Survival Analysis and Verification

Kaplan–Meier survival analysis curves (*p* < 0.05) and a univariate Cox proportional hazards model were used to assess the prognostic value of all biomarkers. GEPIA (http://gepia.cancer-pku.cn/) is a web server for gene expression analyses based on TCGA and GTEx databases (Tang et al., 2017). Using this database to perform differential expression analysis between tumor samples and non-tumor samples. Kaplan–Meier Plotter (http://kmplot.com/analysis/) is an online server that can access the effects of genes on survival in multiple cancer types (Nagy et al., 2021). The statistical significance of expression analyses and survival analysis is defined as *p* < 0.05. Differential expressions of these hub genes at the transcription level were inspected by matching pancreatic tumors and adjacent normal pancreatic tissues from the microarray data we selected in the GEO database. To verify our results, We used the Human Protein Atlas (HPA) online database (www.proteinatlas.org/) to determine the expression of these genes at the translational level (Thul et al., 2017).

### Construction of the ceRNA Network

According to the ceRNA hypothesis, we constructed ceRNA networks by backward prediction. First, we used multiple target gene prediction programs to predict the upstream binding miRNAs of OS-associated genes, including miRbase, miRNet, starBase, Tarbase, PITA, and RNA22. Only the predicted miRNAs that appeared in more than two programs previously were regarded as potential miRNAs for subsequent analyses. Then, the mRNA–miRNA network was established by Cytoscape, and the CytoHubba plug-in was used to determine the top potential miRNAs. Next, Starbase (http://starbase.sysu.edu.cn/) was used to perform survival analysis and differential expression on miRNAs. The upstream lncRNAs of miRNAs identified earlier were predicted in the Starbase. Finally, expression analysis and survival analysis were performed on the identified lncRNA. *p* < 0.05 was considered statistically significant.

### Cibesort Estimation and Survival Analysis of Key Members of the Immune Cells

CIBERSORT (http://cibersort.stanford.edu/) was utilized to evaluate the 22 kinds of immune cell types in PAAD. The *p*-value < 0.05 was chosen as the criterion to identify the immune-infiltrating cells possibly affected by the expression of OS-associated genes. To identify the interrelation between 22 types of immune cells, we performed a correlation-based heat map analysis between every two immune cells. To identify OS-associated immune cells, we used Kaplan–Meier survival analysis to determine the prognostic value.

### Immune Infiltrate Analysis and Immune Checkpoint Expression Analysis

We used the GEPIA database to identify the interrelation between OS-associated genes and biomarkers of immune cells. To investigate the association between the expression of OS-associated genes and immune checkpoint expression, we applied TIMER (https://cistrome.shinyapps.io/timer/), which is an online server that can analyze immune infiltrates across multiple cancer types (Li et al., 2017).

## Result

### Identification of Differential Genes

**FIGURE 1:**
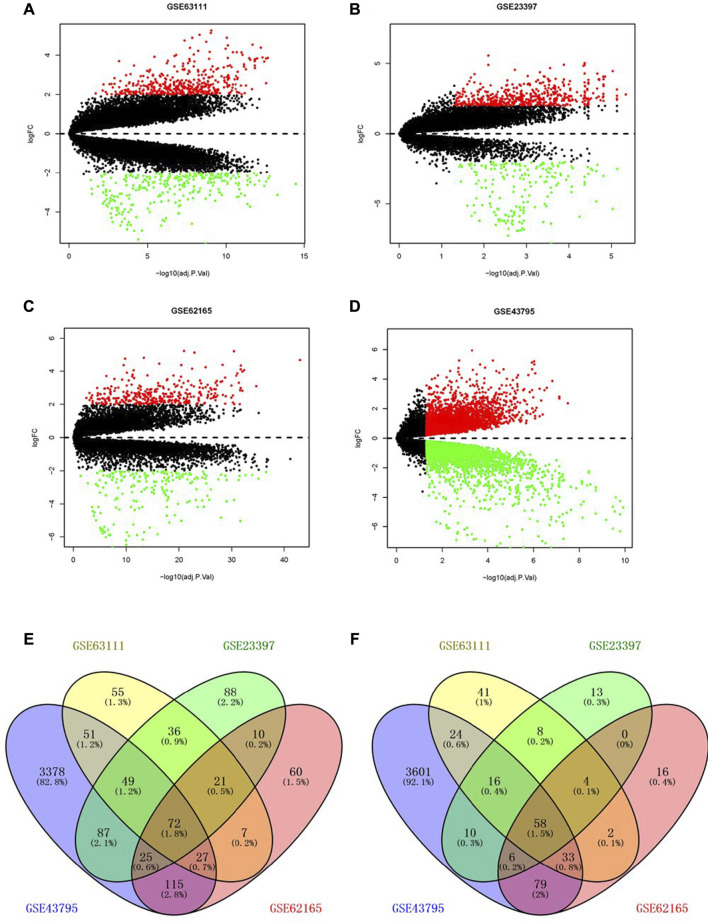
Screening of differentially expressed genes. Volcano plot of GSE63111 **(A)**, GSE23397 **(B)**, GSE62165 **(C)**, and GSE43795 **(D)**. **(E,F)** Upregulated and downregulated DEGs in the GSE63111, GSE23397, GSE62165, and GSE43795. DEGs, differentially expressed genes.

Through the screening of gene expression microarrays associated with PAAD from the GEO database, four datasets (GSE63111, GSE23397, GSE62165, and GSE43795) were chosen. The DEGs in four datasets are shown in Figures 1A–D. The co-expressed DEGs were confirmed by the Venn Diagram online tool, including 72 upregulated and 58 downregulated genes **(** Figures 1D–F).

### Functional Enrichment Analysis of DEGs, and Protein-Protein Interaction Network

**FIGURE 2:**
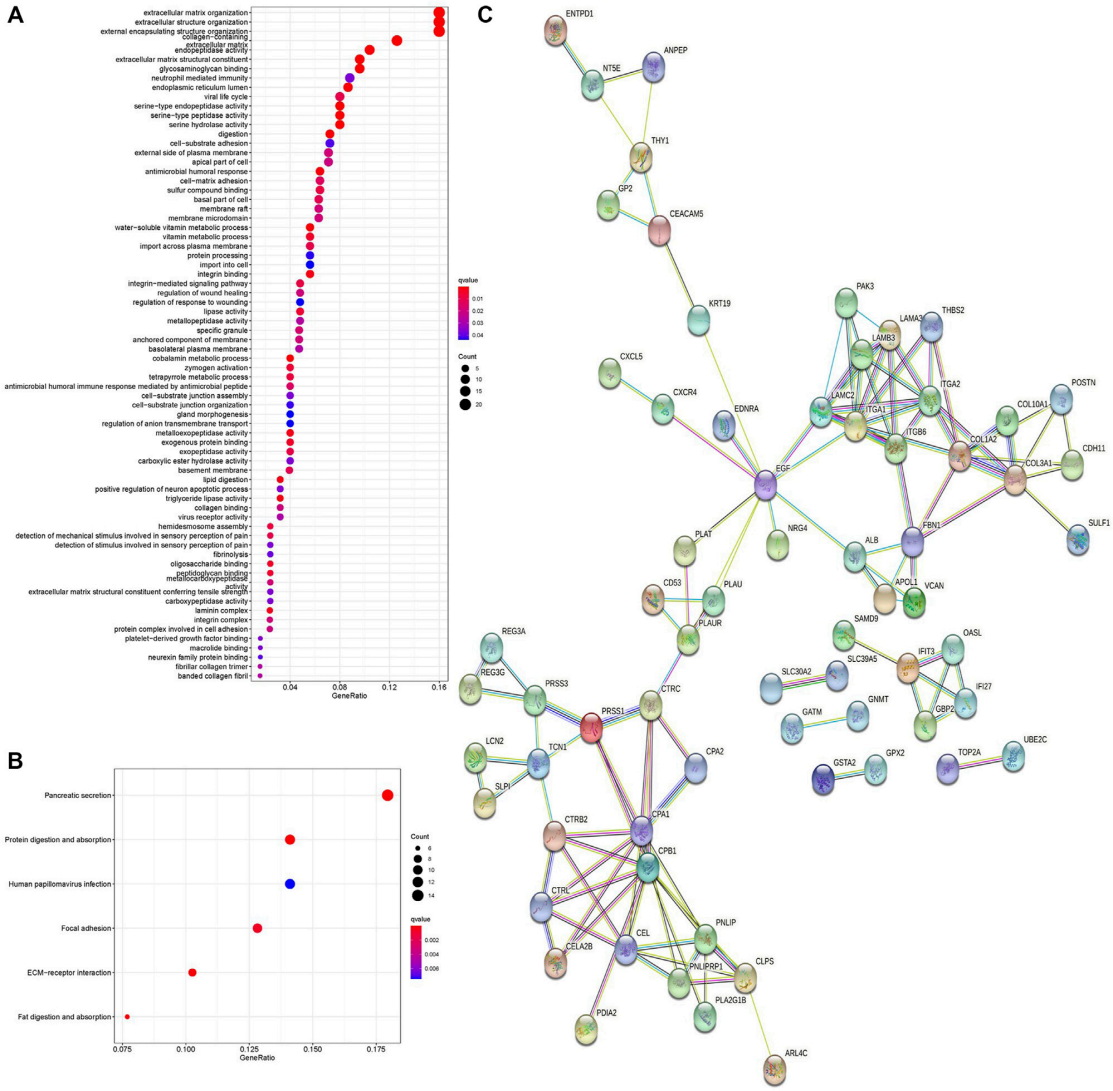
GO enrichment analysis, KEGG pathway analysis, and PPI network of DEGs. **(A)** Bubble chart shows GO enrichment significance items of DEGs. **(B)** Bubble chart shows enrichment of DEGs in signaling pathways. **(C)** PPI network of DEGs. PPI, protein–protein interaction. GO, Gene Ontology. KEGG, Kyoto Encyclopedia of Genes and Genomes.

To further investigate the biological functions of DEGs, we conducted GO and KEGG functional enrichment analyses. In the biological process (BP) group, DEGs were mainly enriched in extracellular matrix organization, extracellular structure organization, and external encapsulating structure organization **(** Figure 2A). In the cellular component (CC) group, DEGs were mainly enriched in the endoplasmic reticulum lumen and collagen-containing extracellular matrix (Figure 2A). In the molecular function (MF) group, DEGs were mainly enriched in endopeptidase activity, extracellular matrix structural constituent, and glycosaminoglycan binding (Figure 2A). Moreover, KEGG pathway analysis results demonstrated that DEGs were significantly enriched in pancreatic secretion and protein digestion and absorption (Figure 2B).

The PPI network of DEGs was analyzed by the STRING online database. A total of 68 nodes and 122 edges were obtained in the PPI network (Figure 2C), and this network was then analyzed using Cytoscape software. Fourteen DEGs were determined as hub genes through the degree algorithm in the CytoHubba plug-in.

### Survival Analysis and Verification

**FIGURE 3:**
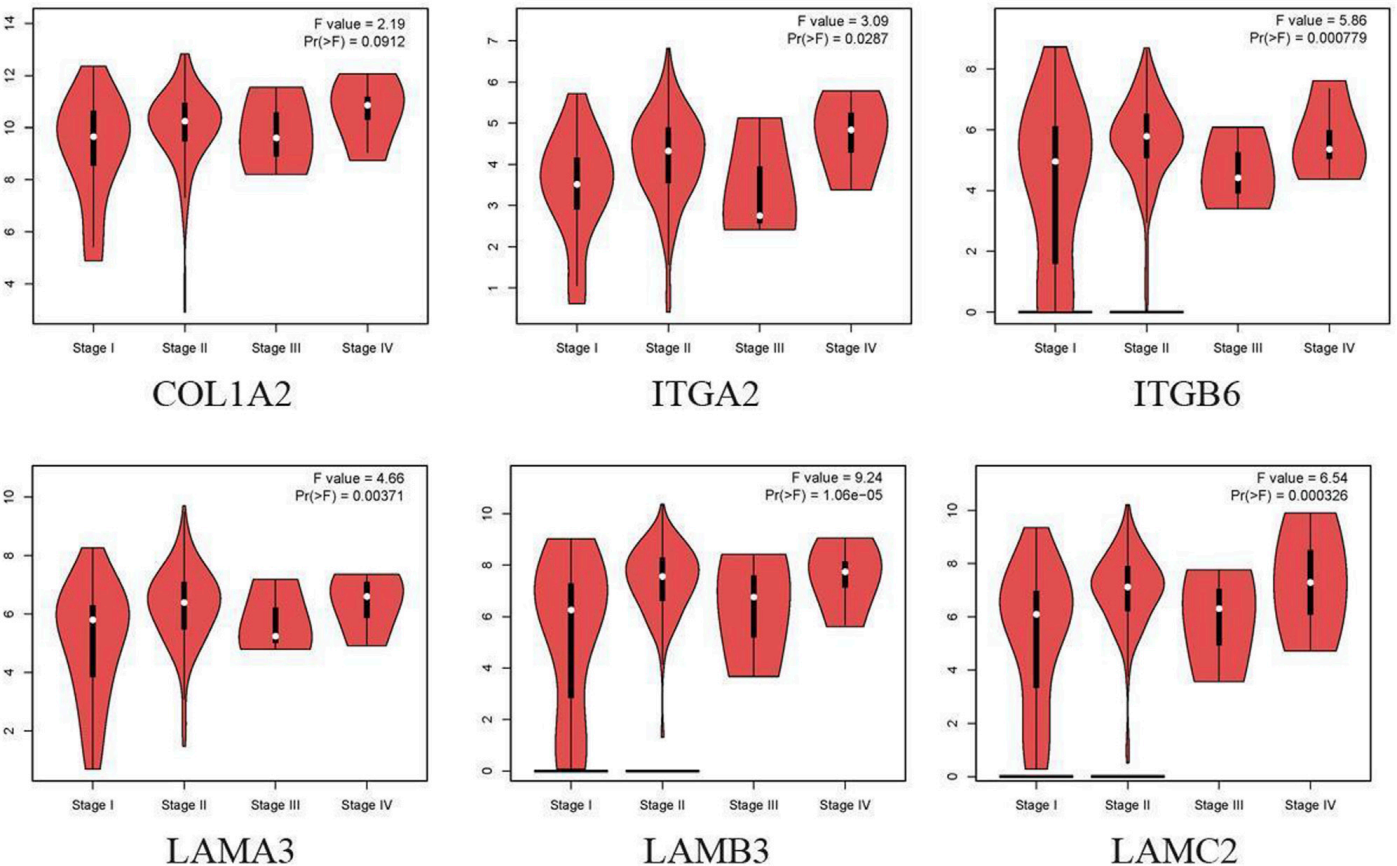
Correlation between OS-related gene expression and tumor stage in PAAD patients (GEPIA).

Cox regression, Kaplan–Meier, and the log-rank test (*p* < 0.05) were used to assess the correlation between DEGs and prognosis. Twenty-five genes were found to be significant in the Kaplan–Meier analysis. However, only six promising prognosis-related biomarkers were hub genes of the PPI network (Table 1). We identified these six genes (COL1A2, ITGA2, ITGB6, LAMA3, LAMB3, and LAMC2) that could obviously affect the OS rate of PAAD patients. We analyzed the expression of OS-related genes with tumor stage for PAAD. The results showed that the expression levels of ITGA2, ITGB6, LAMA3, LAMB3, and LAMC2 are significantly correlated with the tumor stage of PAAD patients, while the expression levels of COL1A2 are not correlated with the tumor stage of PAAD patients (Figure 3).

**TABLE 1:** Cox proportional hazard regression model for OS in patients with PAAD.

Gene	Coef	HR	se (coef)	95%CI_l	95%CI_u	z_score	*P* value
ITGA2	0.335	1.398	0.107	1.134	1.725	3.130971659	0.00174229^**^
COL1A2	0.149	1.161	0.075	1.001	1.346	1.977415373	0.047994698^*^
LAMC2	0.296	1.345	0.081	1.146	1.577	3.635873935	0.00027704^***^
ITGB6	0.309	1.362	0.078	1.170	1.587	3.97318082	7.09E-05^***^
LAMB3	0.269	1.309	0.082	1.115	1.535	3.29746163	0.00097563^***^
LAMA3	0.335	1.398	0.085	1.182	1.652	3.9240794	8.71E-05^***^

**FIGURE 4:**
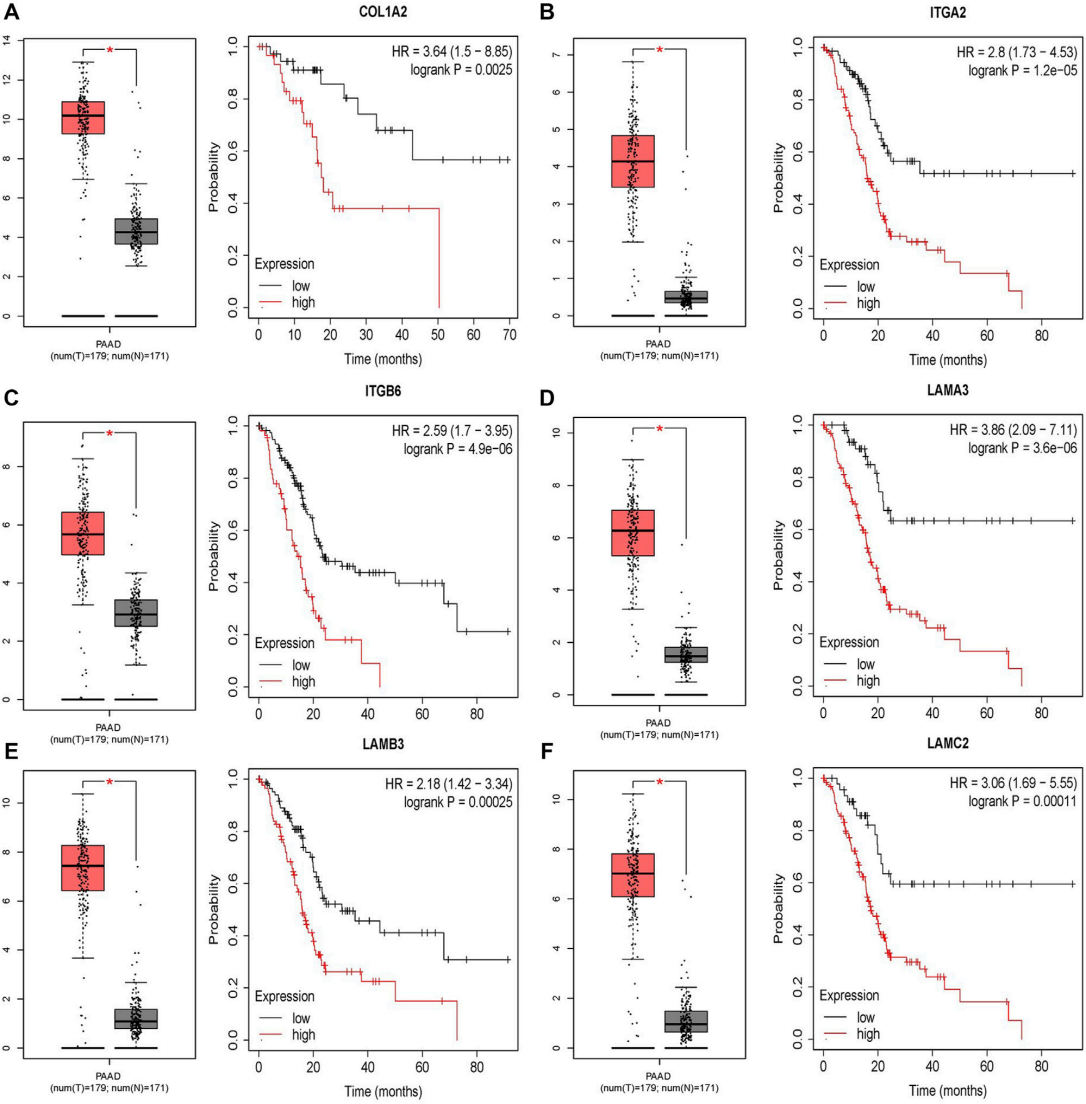
Survival analysis results of six DEGs. **(A–F)** Differential expression and survival analysis of the six selected genes via the GEPIA database. From the abovementioned figures, it was found that these six genes were differentially expressed in the normal group and cancer group and obviously reduce the OS of patients. OS, overall survival.

CI, confidence interval. ^*^ *p* < 0.05, ^**^ *p* < 0.01, and ^***^ *p* < 0.001. In the variable selection process, first of all, the univariate Cox models including all DEGs were used to select potential prognostic genes. At the same time, the Kaplan–Meier analysis was performed based on all DEGs. The results of the Kaplan–Meier analysis (Figures 4A–F) suggested that the six genes have potential prognostic value. Eventually, the univariate Cox model is shown in this table only including six genes filtrating by Kaplan–Meier analysis.

**FIGURE 5:**
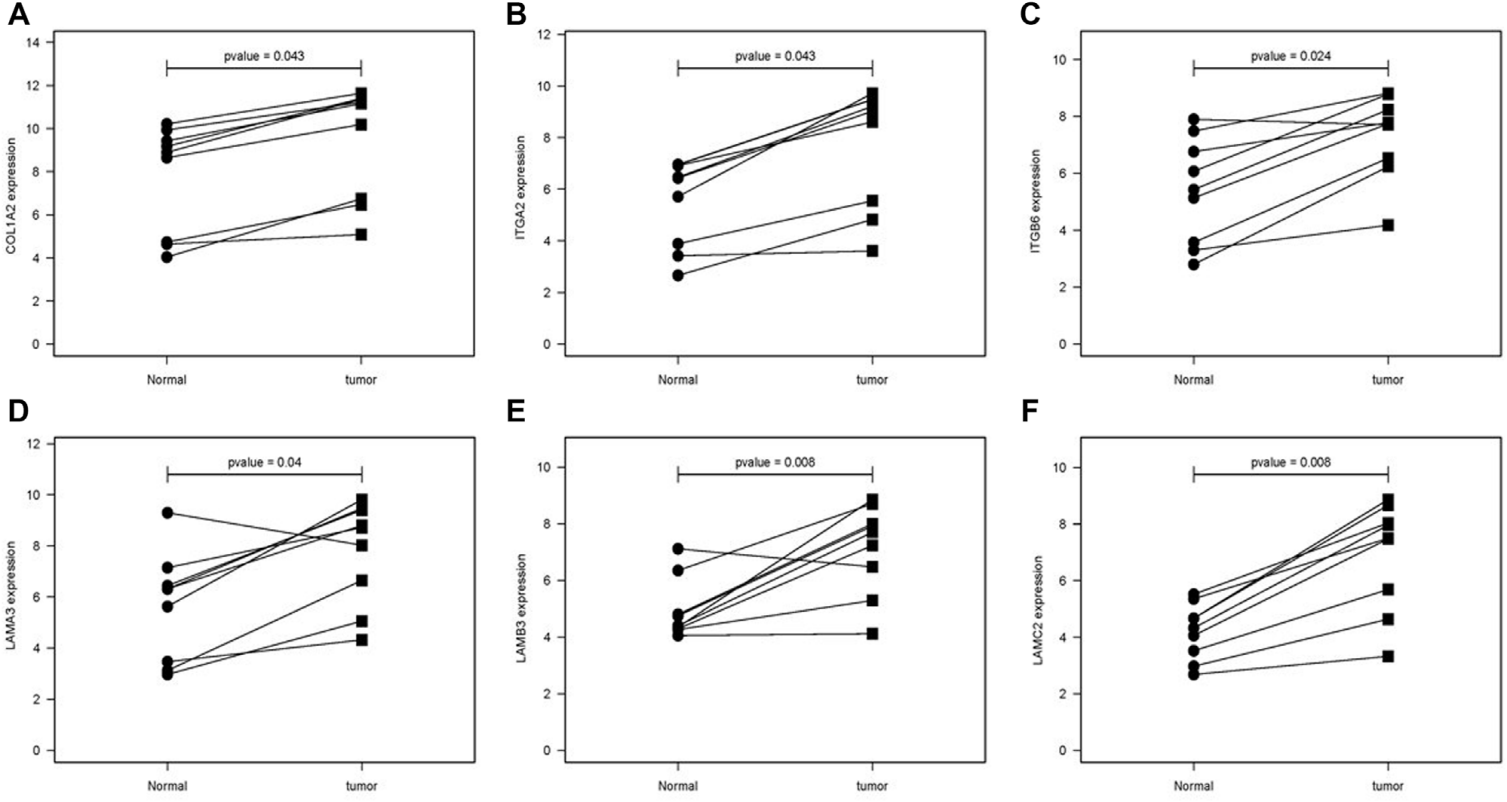
Comparison of the hub gene mRNA levels in paired adjacent normal tissues and PAAD tissues from the GEO database. **(A)** COL1A2, **(B)** ITGA2, **(C)** ITGB6, **(D)** LAMA3, **(E)** LAMB3, and **(F)** LAMC2. PAAD, pancreatic adenocarcinoma.

**FIGURE 6:**
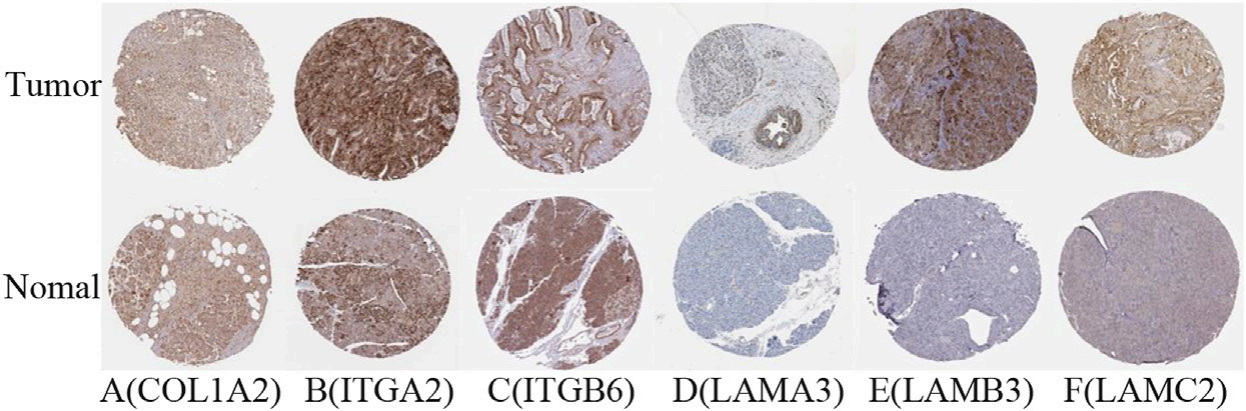
Verification of hub DEG expression in PAAD and normal tissue using the HPA database. **(A)** COL1A2, **(B)** ITGA2, **(C)** ITGB6, **(D)** LAMA3, **(E)** LAMB3, and **(F)** LAMC2.

The difference between the tumor samples and the non-tumor samples was statistically significant (Figures 4A–F). We used R software to analyze the expression of six hub genes at the transcriptional level. The results showed that the expression of COL1A2, ITGA2, ITGB6, LAMA3, LAMB3, and LAMC2 in PAAD tissue was higher than that in adjacent normal pancreatic tissues (Figures 5A–F). Subsequently, we used the HPA database to explore the expression of the hub gene in protein at the translational level. The results illustrated that the protein level of COL1A2, ITGA2, ITGB6, LAMA3, LAMB3, and LAMC2 was higher in PAAD tissues than in normal pancreatic tissues, matching their mRNA expression levels (Figures 6A–F).

### Construction of the ceRNA Network

**FIGURE 7:**
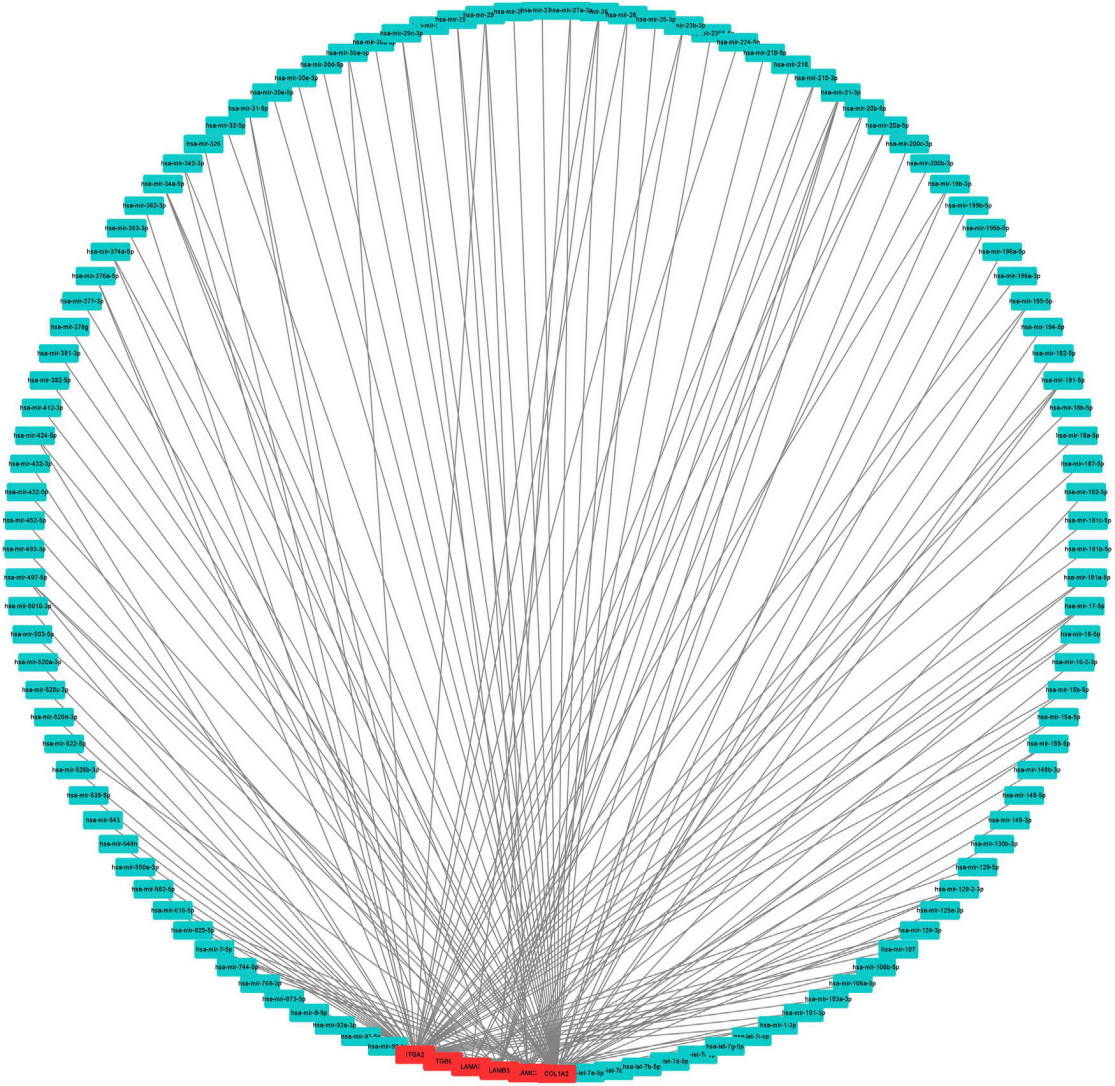
Result of the use of mRNA to reverse-predict miRNA. Red represents highly expressed mRNA, and blue represents miRNA.

**FIGURE 8:**
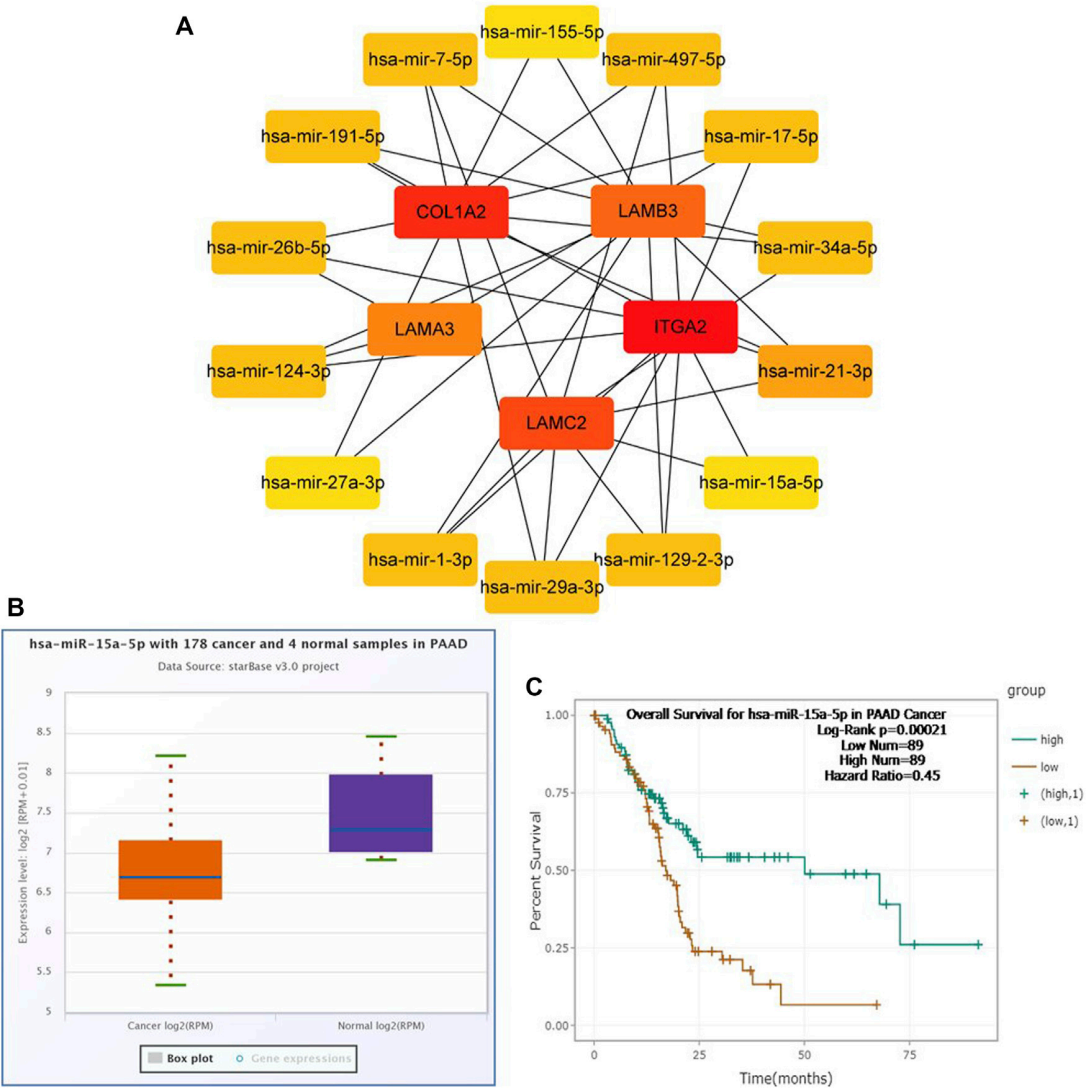
Identification of hsa-miR-15a-5p as a potential miRNA in PAAD. Cytoscape software was used to screen the miRNAs that were closely related to hub-mRNAs. Through the differential expression and survival analysis of the starbase database, one miRNA was selected. **(A)** miRNA–mRNA regulatory network was set up by Cytoscape software. **(B)** Expression of hsa-miR-15a-5p in PAAD and normal samples was examined by the starBase database. **(C)** Prognostic value of hsa-miR-15a-5p in PAAD was evaluated by the Kaplan–Meier plotter.

**FIGURE 9:**
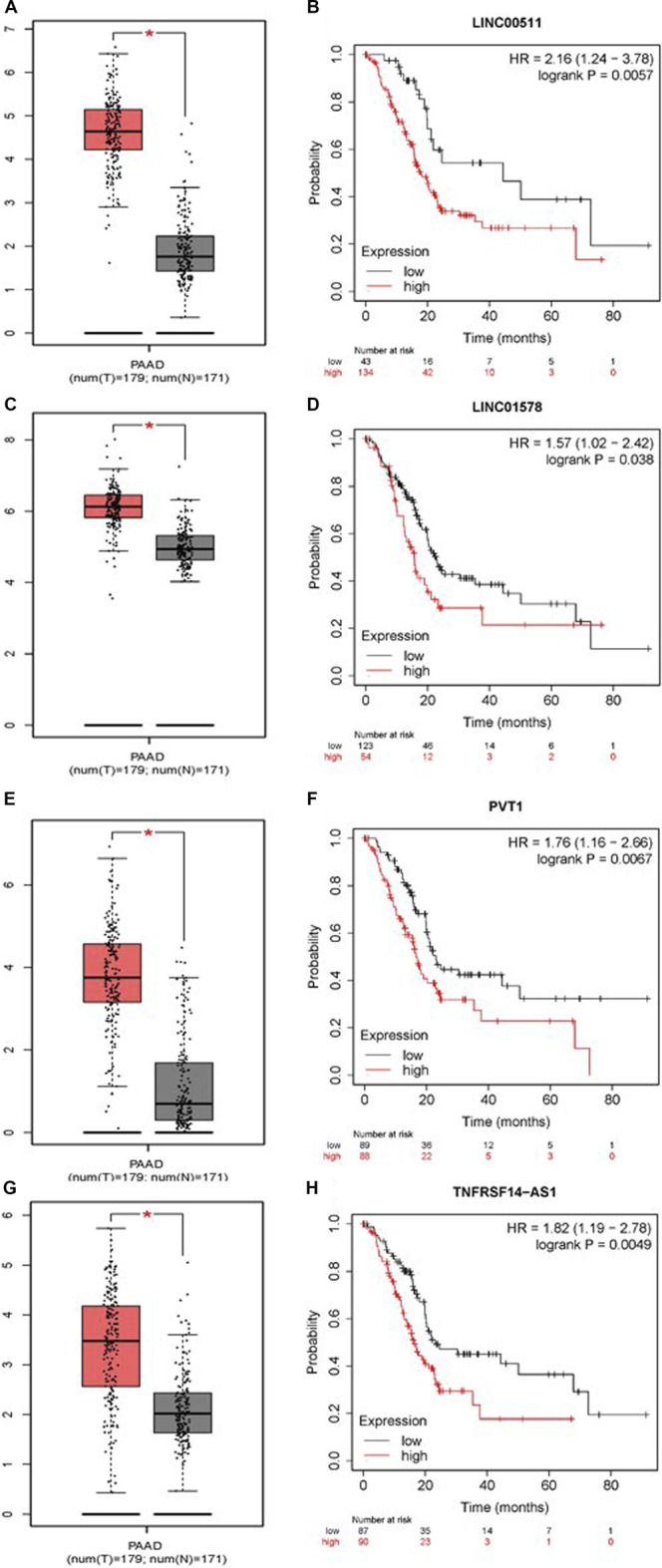
Continued.

To construct a prognostic ceRNA in PAAD, we first predicted upstream miRNAs that could potentially bind to six mRNAs with prognostic values and finally found 160 miRNAs (Figure 7). Cytohubba identified the top fourteen highly connected miRNAs (Figure 8A). The starBase database identified one miRNA, hsa-miR-15a-5p, which was obviously downregulated in PAAD, and its up-regulation was positively correlated with patient prognosis (Figures 8B,C). Next, we use the starBase database to predict the upstream lncRNAs of hsa-miR-15a-5p. 286 possible lncRNAs were obtained. Then, the expression levels of these lncRNAs in PAAD were identified by GEPIA. As shown in Figure 9, LINC00511, LINC01578, PVT1, and TNFRSF14-AS1were significantly upregulated in PAAD compared with normal controls (Figures 9A,C,E,G). Overexpressed LINC00511, LINC01578, PVT1, and TNFRSF14-AS1 indicated poor OS in patients with PAAD (Figures 9B,D,F,H).

### Composition and Co-Expression Analysis of the Immune Cells in Pancreatic Adenocarcinoma

**FIGURE 10:**
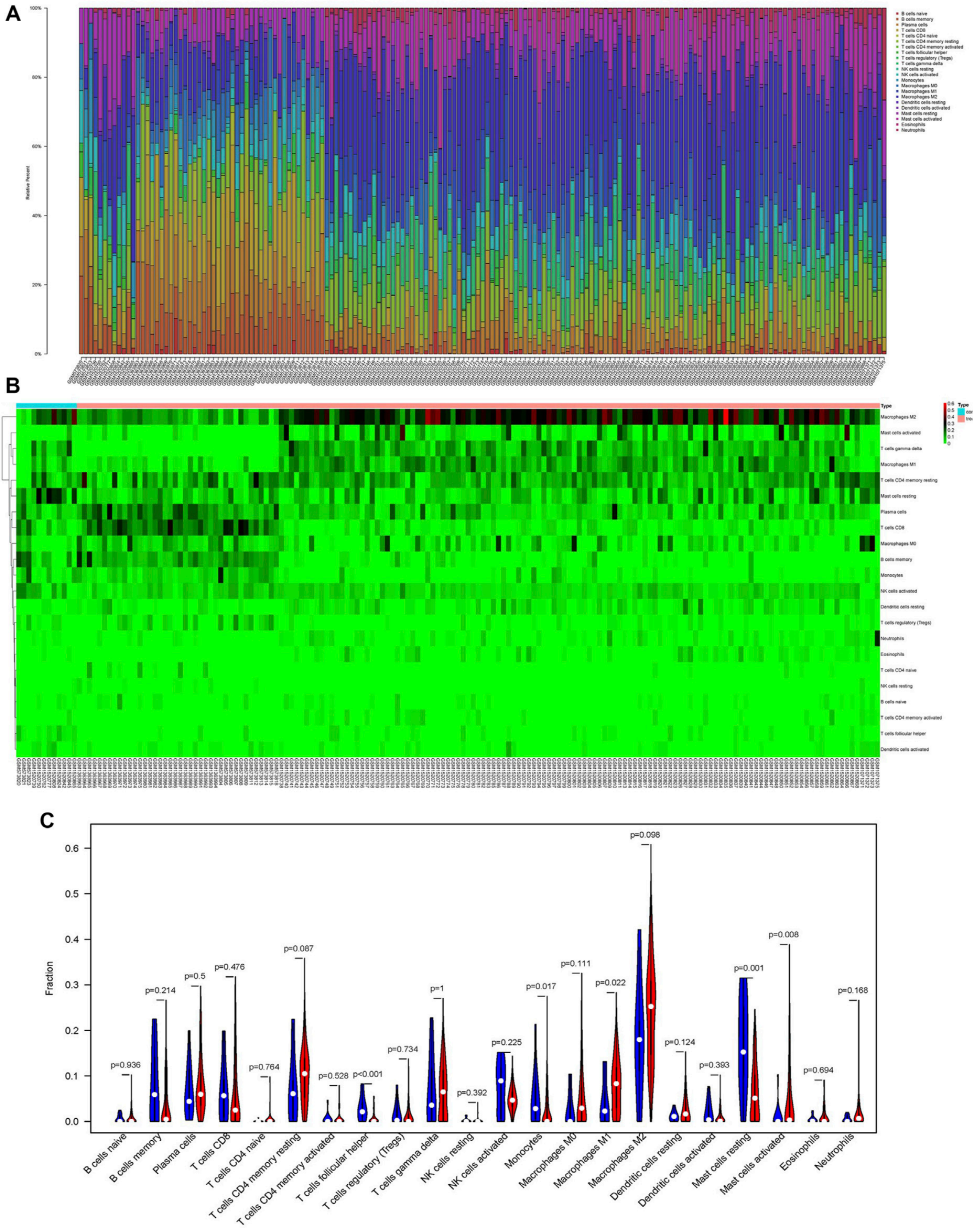
Proportion of the 22 immune cells detected by the CIBERSORT algorithm **(A,B)**. Results of the Wilcoxon rank-sum test **(C)**. Proportions of follicular helper T cells (*p* < 0.001), monocytes (*p* = 0.017), M1 macrophages (*p* = 0.022), resting mast cells (*p* = 0.001), and activated mast cells (*p* = 0.008) were significantly different between pancreatic cancer samples and normal pancreatic samples.

**FIGURE 11:**
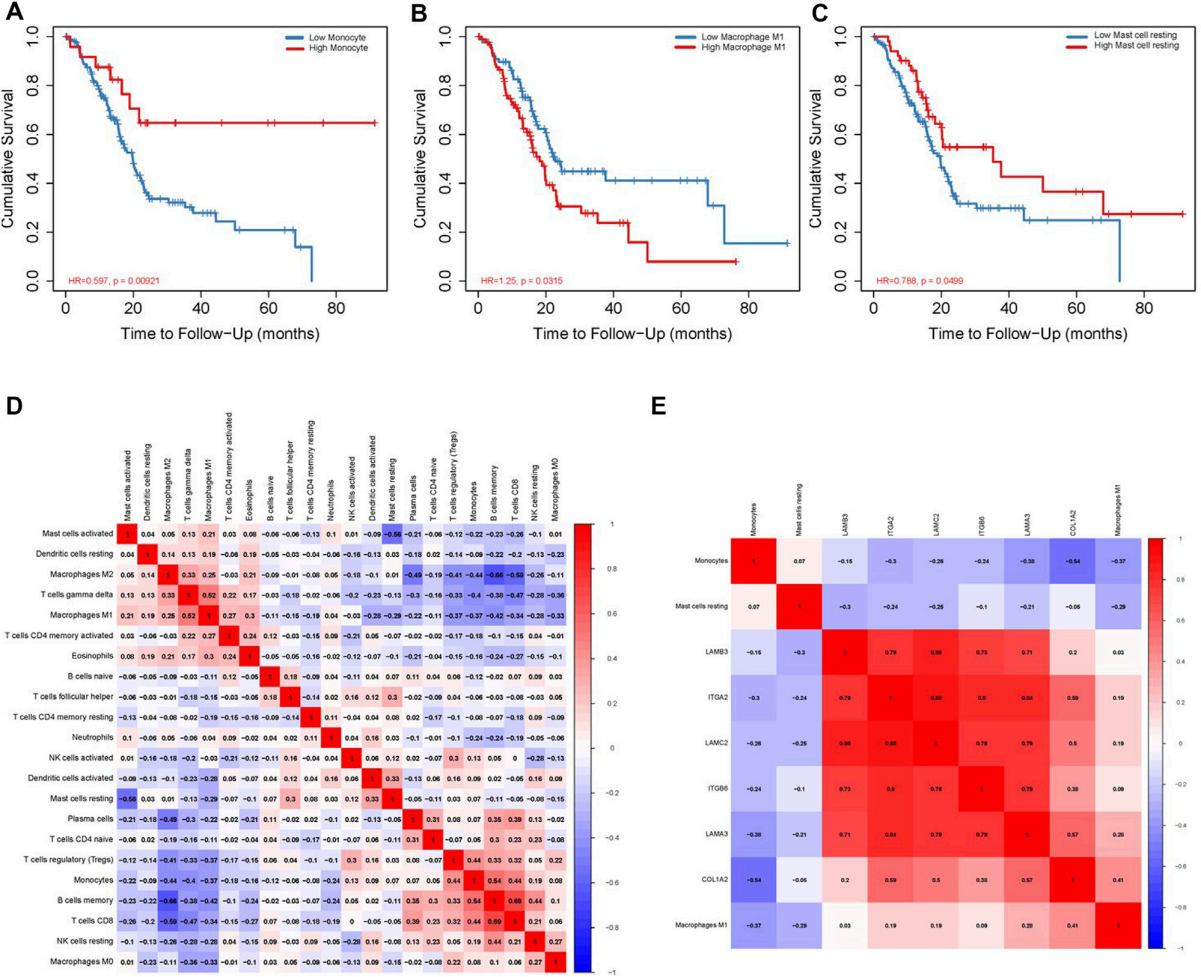
Correlation between immune cell fraction and OS **(A–C)**. Significant co-expression patterns between proportions of immune cells **(D)**. Significant co-expression patterns between hub mRNAs and immune cells **(E)**.

We use the CIBERSORT algorithm to evaluate the composition of tumor-infiltrating immune cells in PAAD tissue. The histogram (Figure 10A) and the heat map (Figure 10B) illustrate the proportions of the 22 immune cells. The violin plot (Figure 10C) reveals the results of the Wilcoxon rank-sum test. The data showed that the fraction of follicular helper T cells (*p* < 0.001), monocytes (*p* = 0.017), M1 macrophages (*p* = 0.022), resting mast cells (*p* = 0.001), and activated mast cells (*p* = 0.008) was obviously different between the pancreatic adenocarcinoma samples and normal pancreatic samples. Then, we used Kaplan–Meier analysis to assess the correlation between the different immune cell subtypes and prognosis. The fraction of monocytes (*p* = 0.00921), M1 macrophages (*p* = 0.0315), and resting mast cell (*p* = 0.0499) was found to be obviously associated with OS (Figures 11A–C). We perform co-expression analysis on prognostic tumor-infiltrating immune cells. (Figure 11D). Finally, Figure 11E showed the relationship of co-expression between hub genes and immune cells. The fraction of M1 macrophages was positively correlated with COL1A2 expression (R = 0.41, *p* < 0.001) (Supplementary Figure S1A). The M1 macrophages were positively correlated with LAMA3 expression (R = 0.28, *p* < 0.001) (Supplementary Figure S1B). The monocytes were negatively correlated with LAMA3 expression (R = −0.38) **(** Supplementary Figure S1C). The monocytes were negatively correlated with ITGA2 expression (R = −0.3, *p* < 0.001) (Supplementary Figure S1D).

### Association of Biomarkers of Immune Cells and Immune Checkpoints With OS-Associated Gene Expression in PAAD

**FIGURE 12:**
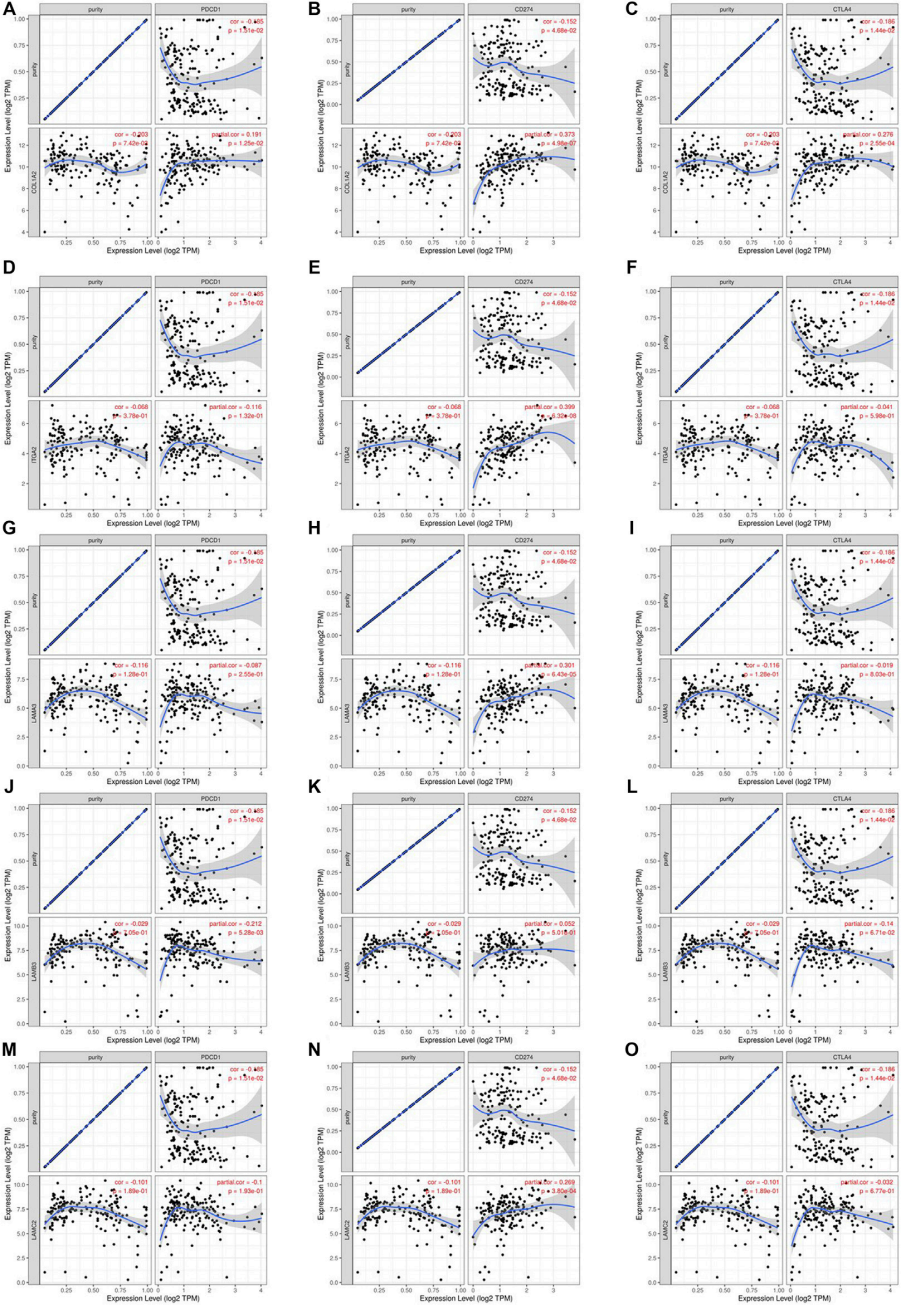
Correlation of hub mRNA expression with PD-1, PD-L1, and CTLA-4 expression in PAAD. Spearman relationship of COL1A2 with an expression of PD-1 **(A)**, PD-L1 **(B)**, and CTLA-4 **(C)** in PAAD adjusted by purity using TIMER. Spearman relationship of ITGA2 with an expression of PD-1 **(D)**, PD-L1 **(E)**, and CTLA-4 **(F)** in PAAD adjusted by purity using TIMER. Spearman relationship of LAMA3 with an expression of PD-1 **(G)**, PD-L1 **(H)**, and CTLA-4 **(I)** in PAAD adjusted by purity using TIMER. Spearman relationship of LAMB3 with an expression of PD-1 **(J)**, PD-L1 **(K)**, and CTLA-4 **(L)** in PAAD adjusted by purity using TIMER. Spearman relationship of LAMC2 with an expression of PD-1 **(M)**, PD-L1 **(N)**, and CTLA-4 **(O)** in PAAD adjusted by purity using TIMER.

We researched the relationship between the expression of OS-associated genes and biomarkers of immune cells. Monocytes, M1 macrophages, and resting mast cells are the significant tumor-infiltrating immune cell. The results were strongly correlated with OS-associated immune cells (Table 2). PD1/PD-L1 and CTLA-4 are significant immune checkpoints for immune escape. We evaluated the relationship of OS-associated genes with PD1, PD-L1, and CTLA-4 by the TIMER platform. As suggested in Figure 12, OS-associated gene expression was clearly positively associated with PD1, PD-L1, and CTLA-4 in PAAD, which was adjusted by purity. These data indicate that immune escape might be concerned with the key gene–mediated poor prognosis of PAAD.

**TABLE 2:** Relationship between hub genes and biomarkers of immune cells in PAAD detected by GEPIA.

Immune Cells	Biomarker	COL1A2		ITGA2		ITGB6		LAMA3		LAMB3		LAMC2	
		R Value	P Value	R Value	P Value	R Value	P Value	R Value	P Value	R Value	P Value	R Value	P Value
Monocytes	CD86	0.33^a^	3.7E-06^***a^	0.16^a^	0.033^*a^	0.34^a^	1.1E-10^***a^	0.29^a^	2E-08^***a^	0.32^a^	1.5E-09^***a^	0.38^a^	1.2E-13^***a^
	CD115(CSF1R)	0.26^a^	0.00048^***a^	0.42^a^	2.2E-16^***a^	0.33^a^	3.7E-10^***a^	−0.16^a^	0.028^*^ ^a^	0.23^a^	1E-05^***a^	0.33^a^	2.4E-10^***a^
M1 macrophages	NOS2	0.3^a^	3.2e-05^***a^	0.21^a^	7.6e-05^***a^	0.092	0.087	0.12^a^	0.022^*^ ^a^	0.17^a^	0.0018^**a^	0.15^a^	0.0063^**a^
	PTGS2	0.15^a^	0.037^*^ ^a^	0.28^a^	0.00012^***a^	0.27^a^	0.00029^***a^	0.19^a^	0.011^*^ ^a^	0.29^a^	8.7E-05^***a^	0.35^a^	1.6E-06^***a^
	IRF5	0.25^a^	6E-04^***a^	0.12	0.11	0.36^a^	2.4E-12^***a^	0.36^a^	3.2E-12^***a^	0.079	0.12	0.4^a^	4.4E-15^***a^
Resting mast cell	CD117(KIT)	0.22^a^	4.9E-05^***a^	0.21^a^	0.0035^**a^	0.24^a^	3.6E-06^***a^	0.18^a^	7E-04^***a^	0.15^a^	0.0058^**a^	0.22^a^	4.9E-05^***a^
	CD203c (ENPP3)	0.18^a^	0.0016^*^ ^a^	0.14^a^	0.0098^**a^	−0.15^a^	0.045^*^ ^a^	−0.23^a^	0.0014^**a^	−0.31^a^	2.4E-05^***a^	0.082	0.12
	SLC18A2	0.27^a^	0.00019^***a^	0.17^a^	0.0016^**a^	0.1	0.051	0.054	0.13	−0.23^a^	0.0016^**a^	0.12^a^	0.025^*a^

a These results are statistically significant.

*p value < 0.05; **p value < 0.01; ***p value < 0.001.

## Discussion

Pancreatic cancer is the deadliest form of cancer. Despite different types of treatment options that have been adopted for pancreatic cancer patients, the prognosis is still poor (Siegel et al., 2020). Molecularly targeted therapies targeting oncogenic genes associated with pancreatic cancer genesis and development are increasingly considered a promising way to cure pancreatic cancer (Paulson et al., 2013). Therefore, illustrating the molecular mechanisms of the pathogenesis of pancreatic cancer and determining potential biomarkers are crucial to improve patient outcomes.

Our study first performed bioinformatics analysis based on GEO datasets, and 130 genes of differentially expressed genes were selected by analyzing the microarray (GSE63111, GSE23397, GSE62165, and GSE43795) related to pancreatic cancer, of which 72 were upregulated and 58 were downregulated. Enrichment analysis showed that DEGs were mainly concentrated in external encapsulating structure organization, extracellular structure organization, and extracellular matrix organization. Next, six key genes in the PPI network were screened by Cox regression and Kaplan–Meier analysis. According to the ceRNA hypothesis, we gradually determined upstream miRNAs and lncRNAs from mRNAs and finally successfully established the ceRNA network. All genes of the ceRNA network are associated with poor prognosis of PAAD. In addition, we found that hsa-miR-15a-5p and hub genes in ceRNA and immune cell subtypes (monocytes, M1 macrophages, and resting mast cell) could predict prognosis effectively. Finally, our study found that high expression of OS-associated genes was closely related to PD1, PD-L1, or CTLA-4 in PAAD, suggesting that targeting mRNAs might improve the efficacy of immunotherapy in PAAD.

COL1A2 belongs to the family of ECM proteins. The ECM provides structural support, mediates cell-matrix adhesion, and integrates complex, multivalent signals for cells (Egusa et al., 2007; Hynes, 2009). Wu et al. Wu et al. (2019a) reduced the expression of COL1A2 to reduce the invasion and migration ability of pancreatic cancer cells and found that COL1A2 was highly expressed in pancreatic cancer patients (*p* < 0.05), which was related to poor prognosis in these patients. ITGA2 belongs to the integrin alpha chain family. Integrin is a membrane protein with cell adhesion and signal transduction functions (Desgrosellier and Cheresh, 2010). Studies have found that the expression of ITGA2 is an independent prognostic marker of PAAD and is related to the grading and aggressiveness of PAAD (Shang et al., 2019; Yan et al., 2020). They also found that the expression of ITGA2 could significantly reduce the survival rate of PAAD patients, which is in accordance with our research. ITGB6 belongs to a family of β2 integrins. Some studies have illustrated that the expression of ITGB6 is negatively related to prognosis in PAAD patients (Wu et al., 2019b), which is consistent with our survival analysis. LAMA3, LAMB3, and LAMC2 belong to the laminin gene family. Laminin is known to stimulate cell migration in cancers (Tripathi et al., 2008). Yang et al. Yang et al. (2019) found that four subunits of the laminin gene family (LAMA3, LAMA4, LAMB3, and LAMC2) were connected with the outcomes of PAAD patients. The findings in our study are consistent with previous reports as we found that the LAMA3, LAMB3, and LAMC2 genes were upregulated in patients with PAAD, with hazard ratios of 3.86, 2.18, and 3.06, respectively. Combined with patient survival curves, these genes were found to be poor prognostic markers in patients with PAAD.

Hsa-miR-15a-5p is a microRNA whose family has already become promising cancer-specific biomarkers (Shao et al., 2020). Compared with PAAD patients with high miR-15a-5p expression, those patients with low miR-15a-5p levels have a shorter overall survival (Guo et al., 2020), which is consistent with the results of our Figure 8C. Functionally, exogenous miR-15a-5p expression decreases cell growth, epithelial-to-mesenchymal transition, and metastasis and increases cell cycle arrest in PAAD (Guo et al., 2014; Feng et al., 2019). Mechanistically, It has been confirmed that miR-15a-5p exerts its anti-pancreatic cancer activity by directly targeting FGFR1, which is highly expressed and executes cancer-promoting actions in PAAD (Turner et al., 2008; Lehnen et al., 2013; Coleman et al., 2014). A study focusing on the mechanism of hsa-miR-15a-5p in pancreatic cancer independent from the CERS6-AS1 (Shen et al., 2021). In addition, research shows a relationship between patient prognosis and tumor-associated macrophage infiltration in pancreatic cancer (Di Caro et al., 2016). In our present study, M1 macrophages which were chosen from CIEBERSORT and the co-expression correlation of COL1A2, LAMA3, and M1 macrophages. The correlation analysis results were consistent with the previous research that indicated that there is a regulatory connection between M1 macrophages and COL1A2 (Zhang et al., 2020b). Cells of the monocyte lineage contribute to tumor angiogenesis (Dalton et al., 2014). One study suggested monocytes are recruited into Pancreatic Ductal Adenocarcinoma tumors via IL35 and increased expression of genes that promote angiogenesis in IL35 products (Huang et al., 2018). Nevertheless, there are few research studies before about the connections of monocytes and Hsa-miR-15a-5p. Thus, we deduced that Hsa-miR-15a-5p, as a miRNA, might connect with monocytes and took active part in the progression of PAAD.

Mishra et al.s’ study represents the first TCGA-based PDAC methylome data analysis and first reported that several genes (B3GNT3, DMBT1, and DEPDC1B) and lncRNAs (PVT1, and GATA6-AS) are strongly correlated with pancreatic ductal adenocarcinoma survival (Kumar Mishra et al., 2019). The conclusions regarding PVT1 are consistent with our results in Figure 9E, F. Raman et al. identified a 5-gene panel (ADM, ASPM, DCBLD2, E2F7, and KRT6A) that performed well against previous signatures across multiple datasets and captures subtype-specific differences in patient prognosis (Raman et al., 2018). This study provides a framework for similar assessments in other cancers.

It should be acknowledged that our study has some unavoidable limitations. First, the quantity of samples we gather from GEO databases is indeed limited, which suggests that clinico pathological information was not comprehensive and might bring about some potential errors or biases. In addition, only a limited number of links were predicted. The ceRNA network is highly complicated and diverse, and its complex regulatory network needs to be further elucidated. Finally, we did not consider the heterogeneity of the immune microenvironment which is allied to the immune infiltration location. The heterogeneity and complexity of the immune microenvironment may affect the generalization of results. However, to reduce the bias, we used numerous databases to reveal the expression of crucial biomarkers at the gene level, protein level, and tissue levels, and it showed that crucial biomarkers were upregulated in PAAD. This study provides valuable material for further research, in which we would further explore the expression levels of key members of the ceRNA network in clinical samples and the interaction mechanism between ceRNA and cell communication through experiments.

In summary, through the integration of several bioinformatics analyses, we constructed a reverse mRNA model based on the LINC00511, LINC01578, PVT1, TNFRSF14-AS/hsa-miR-15a-5p/COL1A2, ITGA2, ITGB6, LAMA3, LAMB3, and LAMC2 ceRNA network, which can enhance our understanding of the development of PAAD. HR values of all genes in ceRNA are greater than 1, indicating that they are risk factors for PAAD, so they might be utilized as potential prognostic biomarkers and therapeutic targets for PAAD. Moreover, our study reveals the potential mechanism that hsa-miR-15a-5p regulates COL1A2, ITGA2, and LAMA3 so that monocytes and M1 macrophages are activated, which may have great effects on pancreatic cancer prognosis.

## Data Availability

The datasets presented in this study can be found in online repositories. The names of the repository/repositories and accession number(s) can be found in the article/Supplementary Material.

## Supplementary Material

Supplementary Figure S1 Fraction of M1 macrophage cells was positively associated with COL1A2 expression **(A)** and LAMA3 expression **(B)**. Monocyte cells were positively associated with LAMA3 **(C)** and ITGA2 **(D)**.
