# Supplementary figures and images for: lncRNAs Functioned as ceRNA to Sponge miR-15a-5p Affects the Prognosis of Pancreatic Adenocarcinoma and Correlates With Tumor Immune Infiltration
Source: Front Genet. 2022 Jul 11;13:874667. doi: 10.3389/fgene.2022.874667 (PMC9312832; doi:10.3389/fgene.2022.874667)

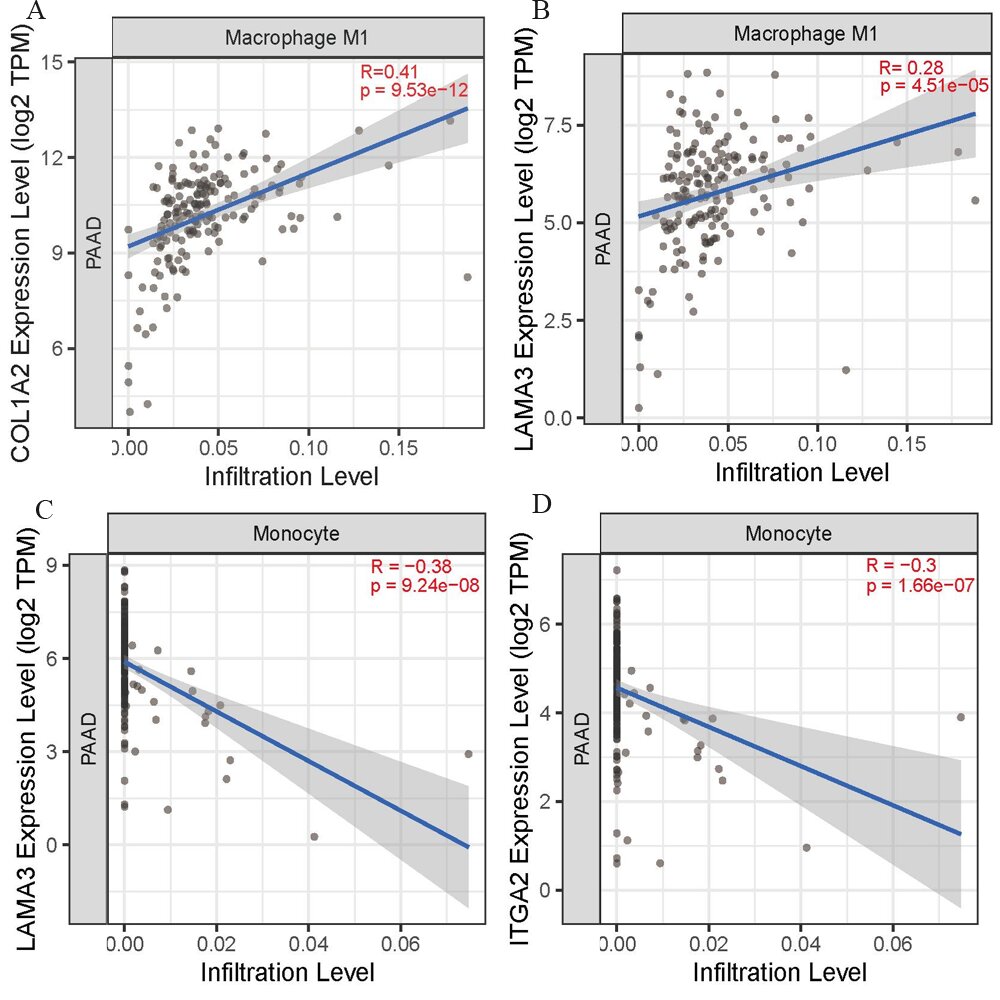

Supplement: Supplementary file 1 [file Image1.JPEG]
