# Supplementary material for: lncRNAs Functioned as ceRNA to Sponge miR-15a-5p Affects the Prognosis of Pancreatic Adenocarcinoma and Correlates With Tumor Immune Infiltration
Source: Front Genet. 2022 Jul 11;13:874667. doi: 10.3389/fgene.2022.874667 (PMC9312832; doi:10.3389/fgene.2022.874667)
Supplement: Supplementary file 2 [file Table1.DOC]

TABLE1|Cox proportional hazards regression model for overall survival in patients with PAAD

| gene | coef | HR | se(coef) | 95%CI_l | 95%CI_u | z_score | P value |
| --- | --- | --- | --- | --- | --- | --- | --- |
| ITGA2 | 0.335 | 1.398 | 0.107 | 1.134 | 1.725 | 3.130971659 | 0.00174229 |
| COL1A2 | 0.149 | 1.161 | 0.075 | 1.001 | 1.346 | 1.977415373 | 0.047994698 |
| LAMC2 | 0.296 | 1.345 | 0.081 | 1.146 | 1.577 | 3.635873935 | 0.00027704 |
| ITGB6 | 0.309 | 1.362 | 0.078 | 1.170 | 1.587 | 3.97318082 | 7.09E-05 |
| LAMB3 | 0.269 | 1.309 | 0.082 | 1.115 | 1.535 | 3.29746163 | 0.00097563 |
| LAMA3 | 0.335 | 1.398 | 0.085 | 1.182 | 1.652 | 3.9240794 | 8.71E-05 |
