# Supplementary material for: lncRNAs Functioned as ceRNA to Sponge miR-15a-5p Affects the Prognosis of Pancreatic Adenocarcinoma and Correlates With Tumor Immune Infiltration
Source: Front Genet. 2022 Jul 11;13:874667. doi: 10.3389/fgene.2022.874667 (PMC9312832; doi:10.3389/fgene.2022.874667)
Supplement: Supplementary file 3 [file Table2.DOC]

|  | coef | HR | se(coef) | 95%CI_l | 95%CI_u | z_score | P value |
| --- | --- | --- | --- | --- | --- | --- | --- |
| Macrophages M1 | 5.956 | 386 | 2.579 | 2.461 | 60557.271 | 2.309 | 0.021 |
| eosinophil | 296.796 | 7.886+128 | 134.523 | 2.46e+12 | 2.53e+243 | 2.206 | 0.027 |
